# Radically open-dialectical behavior therapy for adult anorexia nervosa: feasibility and outcomes from an inpatient program

**DOI:** 10.1186/1471-244X-13-293

**Published:** 2013-11-07

**Authors:** Thomas R Lynch, Katie LH Gray, Roelie J Hempel, Marian Titley, Eunice Y Chen, Heather A O’Mahen

**Affiliations:** 1 School of Psychology, University of Southampton, Highfield Campus, Southampton, UK; 2 Haldon Unit, Wonford Hospital, Devon Partnership Trust, Exeter, UK; 3 Department of Psychology, Temple University, Philadelphia, PA, USA; 4 School of Psychology, University of Exeter, Exeter, UK

**Keywords:** Anorexia nervosa, DBT, Inpatient, Eating disorders, Overcontrol, Personality disorders, Radical openness

## Abstract

**Background:**

Anorexia Nervosa (AN) is a highly life-threatening disorder that is extremely difficult to treat. There is evidence that family-based therapies are effective for adolescent AN, but no treatment has been proven to be clearly effective for adult AN. The methodological challenges associated with studying the disorder have resulted in recommendations that new treatments undergo preliminary testing prior to being evaluated in a randomized clinical trial. The aim of this study was to provide preliminary evidence on the effectiveness of a treatment program based on a novel adaptation of Dialectical Behavior Therapy (DBT) for adult Anorexia Nervosa (Radically Open-DBT; RO-DBT) that conceptualizes AN as a disorder of overcontrol.

**Methods:**

Forty-seven individuals diagnosed with Anorexia Nervosa-restrictive type (AN-R; mean admission body mass index = 14.43) received the adapted DBT inpatient program (mean length of treatment = 21.7 weeks).

**Results:**

Seventy-two percent completed the treatment program demonstrating substantial increases in body mass index (BMI; mean change in BMI = 3.57) corresponding to a large effect size (*d* = 1.91). Thirty-five percent of treatment completers were in full remission, and an additional 55% were in partial remission resulting in an overall response rate of 90%. These same individuals demonstrated significant and large improvements in eating-disorder related psychopathology symptoms (*d* = 1.17), eating disorder-related quality of life (*d* = 1.03), and reductions in psychological distress (*d* = 1.34).

**Conclusions:**

RO-DBT was associated with significant improvements in weight gain, reductions in eating disorder symptoms, decreases in eating-disorder related psychopathology and increases in eating disorder-related quality of life in a severely underweight sample. These findings provide preliminary support for RO-DBT in treating AN-R suggesting the importance of further evaluation examining long-term outcomes using randomized controlled trial methodology.

## Background

Anorexia Nervosa (AN) is a serious psychiatric illness characterized by low body weight and intense fears of gaining weight [1]. In adulthood, the course of AN is frequently chronic, and is characteristically difficult to treat. Rates of mortality in AN are higher than in any other mental disorder, with death primarily resulting from cardiac problems or suicide [2,3]. Naturalistic follow-up studies suggest that less than half of adults with AN improve while the majority continue on chronic courses or only partially improve [4]. For adults with AN, no specific treatment has been shown to be superior, in part because there is a dearth of adequately designed and powered randomized controlled trials (RCTs) [5]. Further, many treatments have failed to adequately attend to the core symptoms of AN [5]. British (NICE, [6]) and US (APA, [7]) guidelines therefore make no specific recommendations for the treatment of AN in adults. Thus, new theoretical and treatment approaches are needed for this disorder.

To date, a number of different psychological treatments for AN have been studied, including family-based therapy (FBT), cognitive-behavioral therapy (CBT), cognitive-analytic therapy (CAT), and non-specific supportive clinical management (NSCM) [8]. The majority of psychological treatments have been tested in outpatient settings [8]. However, based on reviews and meta-analyses, there is no evidence of the superiority of one treatment approach over another [8]. Although family-based approaches have been shown to be effective in younger, non-chronic AN patients [9-11] adult AN patients fare poorly compared to adolescents [12]. Likewise, although there is evidence for the effectiveness of cognitive behavioral approaches in treating Bulimia Nervosa (BN) [13], it has been less successful in the treatment of AN, with equivalent outcomes to NSCM (although this specific study was underpowered) [14]. Using an enhanced version of CBT (CBT-E), a large uncontrolled trial by Fairburn et al. [15]. found significant and large pre-post changes in BMI (mean baseline BMI = 16.0, *SD* = 1.2; mean change in BMI = 1.8). In addition, improvements in psychological functioning were found and maintained at 60-month follow-up. However, this study excluded individuals who had received specialist eating disorder services in the previous year and who were at risk of hospitalization, suggesting there is still a need to investigate treatments for those with severe and or deteriorating courses of AN. Lastly, RCTs investigating the success of outpatient CAT for adult AN have indicated mixed results. In two separate trials, one comparing CAT with an educational-behavioral therapy and the other comparing it to FBT and focal psychotherapy, there were no differences between CAT and the other treatments [16,17]. However, it should be noted that both studies were underpowered to detect treatment differences.

Due to the high medical and psychiatric risks associated with AN and lack of progress in outpatient treatments often necessitates hospitalization, it is important to examine the effectiveness of psychological treatment models used in inpatient settings. There have been few studies and no RCTs examining inpatient treatment for AN. Although Hartmann and colleagues [8] reported an overall effect size of 1.2 in weight gain for inpatient treatments, the overall lack of research on inpatient programs is a critical gap given that those with lower BMIs and more severe, chronic presentations, are more representative of inpatient treatment. Cognitive-Behavioral Therapy, IPT and DBT have been used in some inpatient settings as part of a multi-component approach to treating Anorexia. However, there is a lack of research on the efficacy of these approaches in inpatient settings, and because most inpatient units implement a given treatment as part of a complex treatment package [8], it is often difficult to discriminate which treatments constitute the active components of the service. Thus, given the lack of evidence for existing treatments, there is an acute need to study innovative treatment approaches that are suitable for AN, particularly for those with more severe presentations.

### AN-R and overcontrol: a transdiagnostic perspective

Self-control—inhibiting acting on urges, impulses, and desires—is highly valued in most societies, and failures in self-control characterize many of the personal and social problems afflicting modern civilization. However, too much self-control can be equally problematic. Overcontrol (OC) or excessive inhibitory control has been linked to social isolation, poor interpersonal functioning, hyper-perfectionism, rigidity, risk aversion, lack of emotional expression, and the development of severe and difficult-to-treat mental health problems, such as chronic depression, anorexia nervosa, and obsessive compulsive personality disorder [18-21]. Relatedly, research robustly links eating disorders to three “personality subtypes”: overcontrolled, undercontrolled, and low psychopathology [22]. AN-R (restrictive subtype) is most representative of the overcontrolling subtype, with behavioral patterns paralleling those of other OC disorders (e.g., obsessive compulsive personality disorder), such as; propensities for aloofness/social withdrawal, cognitive rigidity and insistence on sameness, low novelty seeking/insensitivity to reward, strong personal needs for structure and symmetry, heightened threat sensitivity, clinical perfectionism [21,23,24], and invalidating or critical childhood environments [25,26]. Deficits in emotional functioning in AN-R include impaired recognition of emotion in others and reduced emotional expression, particularly the expression of negative emotions [27]. To date, however, this constellation of OC characteristics has not been the primary focus of treatment for AN-R.

### Radically open-dialectical behavior therapy (RO-DBT)

Dialectical behavior therapy (DBT) was originally designed for individuals with borderline personality disorder (BPD) [28,29] and has been shown to be effective in two RCTs targeting binge-purge eating disorders (EDs) with undercontrolled problems such as severe emotion dysregulation [30,31]. To date, there has been no study using standard DBT to specifically target EDs characterized by problems of OC. Lynch and colleague’s adaptation of DBT for OC, referred to as Radically Open-DBT (RO-DBT) [32], has been informed by experimental, longitudinal, and correlational research on overcontrol and related constructs (for a review, see [33]), two RCTs that have focused on OC in chronic/refractory depression [34,35], and mechanisms of change are being evaluated in an on-going multi-center RCT for refractory depression (project REFRAMED; chief investigator: Lynch). Radically open-DBT was developed and conceptualized as a transdiagnostic treatment for disorders of overcontrol such as AN-R.

While resting on many of the core principles of standard DBT, the therapeutic strategies in RO-DBT are often substantially different, both theoretically and practically. For example, RO-DBT contends that *emotional loneliness* represents the core problem for OC, not *emotion dysregulation.* Treatment strategies targeting loneliness and social isolation are informed by a biosocial theory [20,36] positing that OC develops via transactions between temperamental biases for heightened threat and diminished reward sensitivity and family/environmental experiences emphasizing mistakes as intolerable and self-control as imperative. A major component of this theory is that heightened threat sensitivity makes it more difficult for an individual with OC to enter into their neurologically based safety zone [33]. Feeling safe activates the ventral-vagal mediated parasympathetic nervous system (PNS-VVC) associated with contentment, social engagement, and pro-social behaviors via the facial muscles that are involved in maintaining eye contact, listening to human speech and making appropriate facial expressions [37,38]. While the organism feels safe, the PNS-VVC is dominantly active and suppresses the sympathetic nervous system (SNS), allowing the organism to explore and communicate with others [37,38]. Neuroimaging studies support the link between the PNS and feeling safe: the ventromedial prefrontal cortex has been found to modulate the vagal efferent outflow to the heart [39] and promotes safety while inhibiting SNS activity [40,41]. However, when the environment is perceived as threatening, PNS-VVC dominance is withdrawn and the SNS, associated with mobilization behavior (e.g. flight and fight), becomes dominant, increasing heart rate and down-regulating the activation of the striated muscles of the face and head, thus reducing the individual’s ability to engage with the social world [37,38]. This process is linked to activation of the dorsal anterior cingulate cortex, which has been found to promote fear responses via increases in SNS activity [40].

For the OC individual, defensive arousal, frozen or disingenuous expressions and stilted interactions are common; secondary to heightened threat sensitivity and exacerbated by sociobiographic feedback overvaluing self-control and avoidance of criticism. Thus, an OC patient may without conscious awareness exhibit blank facial expressions and long silences on the outskirts of conversation circles, unknowingly scowl when they go to a party, or habitually force smiles or behave in a stilted overly pro-social manner that does not make sense in the current social situation. Unfortunately, masking inner feelings or incongruence between felt experience and displayed behavior makes it more likely for others to perceive the incongruent person as untrustworthy or inauthentic [19,42,43]. In comparison to non-suppressors, habitual suppressors of emotional expression report feeling more inauthentic and greater discomfort with intimacy [44]. Thus, OC self-control efforts, designed to sidestep social difficulties, function to create the very consequences that OC individuals fear the most. That is, people prefer not to interact with them and see them as inauthentic, false, and/or untrustworthy, leading to heightened experiences of social ostracism and loneliness [36].

Consequently, RO-DBT links neurophysiology and the communicative functions of emotion to the formation of close social bonds. As such, a number of treatment strategies are designed to enhance social connectedness, including novel skills to activate PNS-VVC social-safety, signal cooperation (for example, deliberately changing body postures and facial expressions, e.g. leaning back rather than forward and keeping eyebrows up rather than down when stressed), encourage genuine self-disclosure, and break-down over-learned expressive inhibitory barriers (via skills designed to encourage playful behavior and disinhibited expression). Crucially, RO-DBT posits that, for OC patients, it is critical to first engage neurophysiological systems [37,38] that activate social-safety responses and signal cooperation to others prior to engaging in social interactions. In so doing, OC individuals are naturally able to relax facial and nonverbal expression and engage reciprocally in fluid and genuine social interactions [32]. The emphasis on social-signaling and changing neurophysiological arousal in treating OC is key as it differs from other treatments that emphasize interpersonal skills, behavioral experiments, cognitive-restructuring, or ritualized patterns of eating. Once activated, the social-safety system is also hypothesized to reduce compulsive negative affect driven desires to restrict food based on research showing neuro-inhibitory relationships between the ‘calming’ parasympathetic nervous system and the ‘activating’ sympathetic nervous system (SNS) [45].

In addition, RO-DBT conceptualizes restrictive and ritualized eating as a form of maladaptive inhibitory control that has been intermittently reinforced. For example, we posit that, following periods of intense restrictive eating the AN patient’s neuroregulatory system ‘perceives’ the depleted metabolic state as life-threatening; thereby activating the evolutionary ‘older’ parasympathetic nervous system, the dorsal vagal complex (PNS-DVC) [37,38], which functions to inhibit energy depleting SNS-mediated action tendencies, resulting in reduced pain sensitivity and emotional numbing (e.g., flat affect). Thus, we suggest that food restriction and starvation is reinforced because it functions to reduce defensive-arousal secondary to DVC activation. Importantly, this emotion regulation strategy is not only potentially lethal, but flattened and numbed emotional expressions secondary to DVC activation, as reviewed above, are posited to exacerbate social ostracism (see other examples below).

### RO-DBT treatment modes and targets

The functions and modes of RO-DBT are similar to those in standard DBT [28], including weekly 1 hour individual therapy sessions, weekly skills training classes, telephone coaching (as needed), and weekly therapist consultation team meetings. The primary target/goal in RO-DBT is to decrease severe behavioral overcontrol *rather* than decrease severe behavioral dyscontrol as in standard DBT [28].

#### RO-DBT orientation and commitment

The orientation and commitment stage of RO-DBT takes four sessions and can be broken down broadly into four sequential steps: 1) hearing the patient’s story; 2) identifying individualized goals and targets; 3) explaining the therapeutic rationale, and 4) determining willingness and commitment to the treatment. RO-DBT considers it essential for therapists to identify goals and values that are not solely linked to food, weight, body shape, or other similar ED issues when treating AN-R. From the outset RO-DBT therapists ‘smuggle’ the idea to their AN-R patient that they are “much more than an eating disorder”. ‘Smuggling’ refers to an RO-DBT communication strategy designed to introduce new information to an OC patient by ‘planting a seed’ of the idea first using an easy manner. This strategy allows patients the opportunity to reflect on the new information without feeling compelled to accept or reject it immediately. The basic idea is that committing to changing a problem behavior is easier if the patient realizes that the behavior is preventing them from achieving what they value or would like to achieve. Examples of non-ED related goals or values include: *to raise a family, to be gainfully and happily employed, to be more self-aware, to develop or improve close relationships, to establish a romantic partnership, to become better educated.*

During the orientation and commitment period on the inpatient unit in which this study was conducted, participation in the “RO-DBT Program” is voluntary. Patients are given the option to participate in treatment as usual, referred to as the “Engagement Program”, that has less emphasis on psychological factors and more emphasis on weight gain. Contingency management principles are used to facilitate participation in RO-DBT. Specifically, it is explained that since patients in the “RO-DBT program” must work hard to learn new skills, expectations regarding weight gain are more flexible in order to compensate for the additional effort required, whereas this is not the case for the “Engagement Program”.

RO-DBT individual therapy targets are arranged in a hierarchy of importance: 1) reduce life-threatening behaviors, 2) repair alliance-ruptures, and 3) reduce OC maladaptive behaviors linked to common OC themes. The first priority in treatment of OC, similar to standard DBT, is to target the reduction of life-threatening behaviors, defined as: 1) actions, plans, desires, urges, or ideation, the goal of which is to *intentionally* cause tissue damage or death (e.g., non-suicidal self-injury, suicidal ideation/urges, suicide attempt), and 2) behaviors that are not intentionally aimed at dying/tissue damage but are an imminent threat to life. For example, being underweight, restricting, or purging would be considered quality-of-life interfering behavior until the moment a physician says it is imminently life threatening. It is then considered life-threatening behavior (even though the intent is not to damage tissue or cause death), thereby trumping all else except other life-threatening behaviors. The key word to remember in the second part of this definition is “imminent”. This provides a coherent rationale for staff to avoid expressions of over-concern about medical risk when doing so might reinforce dysfunctional behavior. For example, heightened concern about a non-life-threatening low BMI might inadvertently reinforce future restrictive eating or desires to appear medically ill because the additional attention conveys a ‘special status’ to the patient, may excuse a patient from normal expectations or responsibilities, and/or may block work on non-eating disorder issues that may be essential for recovery. As one AN-R patient described it: “*I fear that if I don’t look fragile then I will be ignored or disappear and lose my status as a princess*”. Thus, this approach helps mitigate potential reinforcement of AN-R maladaptive behaviors and allows therapists to attend to psychological issues without concern that they are neglecting medical risk.

Secondly, unlike standard DBT, RO-DBT hierarchically targets *therapeutic alliance-ruptures* over *therapy-interfering behaviors*. This is a major deviation from standard DBT where therapy-interfering behaviors are considered the second most important target in the treatment hierarchy (after life-threatening). Broadly speaking, therapy-interfering behaviors in standard DBT [28] refer to problem behaviors that interfere with the patient receiving the treatment. Common therapy-interfering behaviors in standard DBT might include; non-compliance with diary cards, no-showing for sessions, repeatedly crossing therapists’ personal limits leading to demoralization, or refusal to speak during a session. Thus, therapy-interfering behaviors in standard DBT are problematic behaviors prioritized for change. In stark contrast, alliance-ruptures are not considered problems; they are considered opportunities for growth. Alliance-ruptures are the essential practice grounds to learn how conflict can be intimacy enhancing and a successful alliance-rupture repair blocks overlearned OC tendencies to abandon relationships. Alliance-ruptures are conceptualized to revolve around two issues: 1) the patient feels misunderstood, and/or 2) the patient experiences the treatment as not relevant to their unique issues. When an alliance-rupture is suspected, the therapist should *drop their in-session agenda* (e.g., conducting a behavioral chain analysis) and shift their attention toward the relationship with their patient. Typically this involves *slowing down* the pace of the interaction and directly asking the patient what is happening in the moment (details regarding alliance-rupture repairs are provided in the treatment manual [32]).

#### Targeting maladaptive OC behaviors

**Figure 1:**
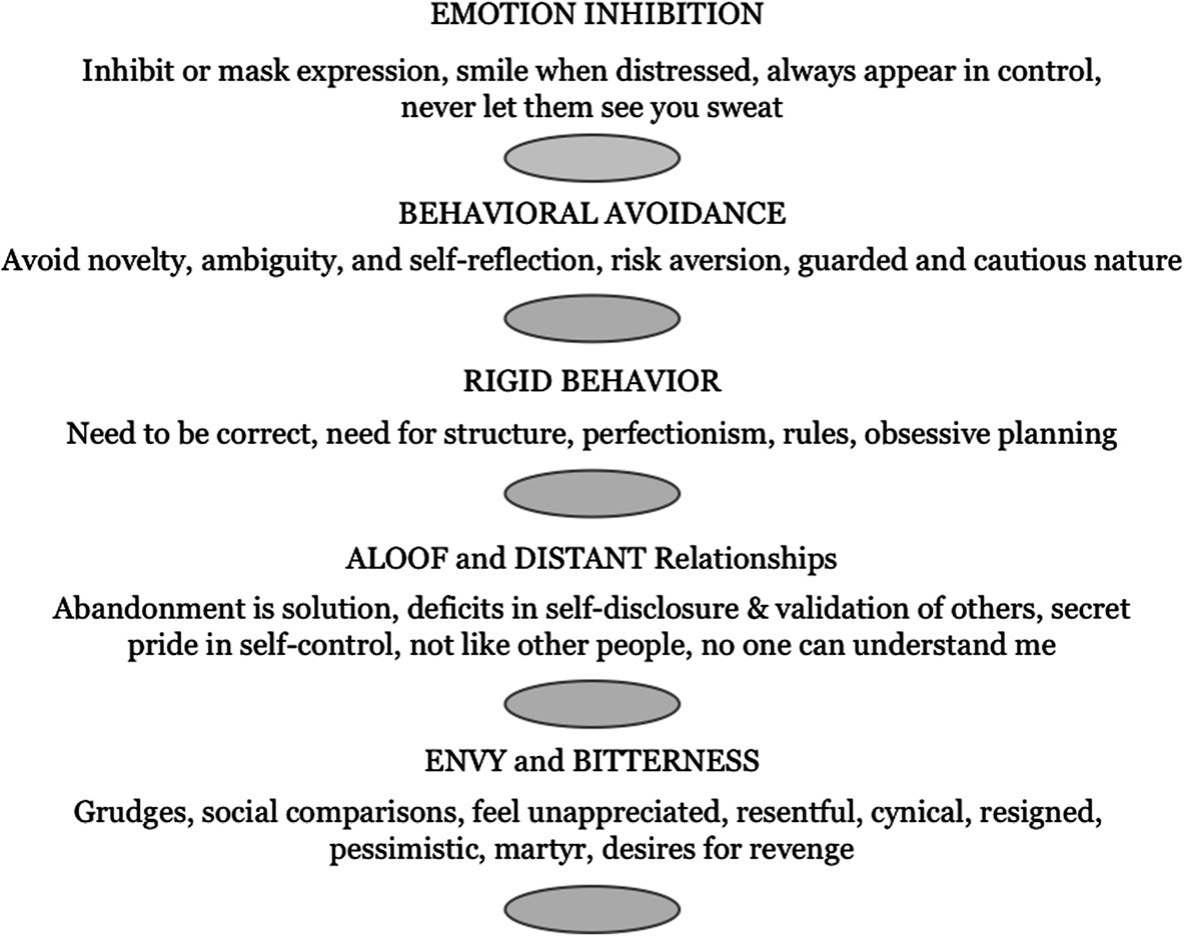
Path to flexible mind: OC behavioral themes.

Though life-threatening and therapeutic alliance-ruptures take precedence when present, the third most important target in the RO-DBT treatment hierarchy centers around the reduction of maladaptive OC behaviors. The Path to Flexible-Mind (see Figure 1) overviews the five most common OC behavioral themes that are used to develop behaviorally specific individualized targets monitored daily on diary cards. Diary cards are used during individual therapy as means for identifying the most serious or problematic behavior occurring in the past week that will form the basis for a behavioral chain and solution analysis (the treatment manual provides details for assessing and treating specific OC behaviors and examples of diary cards [32]). Unless imminently life threatening, RO-DBT for AN-R discusses ED behaviors (e.g., restriction, body shape, exercise) in the latter part of individual therapy sessions (e.g., last 20 minutes). This approach differs from other treatments that prioritize ED behaviors over other problems, and is informed by: 1) robust research linking AN-R to overcontrolled problems that pre-existed the diagnosis of AN-R, 2) a trans-diagnostic philosophy that underlies RO-DBT positing that disorders of overcontrol are best treated when maladaptive OC behaviors are given priority, and 3) clinical observations that excessive attention directed toward intractable ED cognitions/behaviors may function to inadvertently reinforce the maladaptive behavior and/or may block discussion of other important life issues. As one AN-R patient put it: “*When my therapist focuses on my ED behaviors I feel a sense of relief—talking about ED is a lot easier than facing reality*”. The goal is to target ED behaviors without unnecessarily reifying them and/or inadvertently reinforcing them by making them the sole focus of treatment. Consequently, the RO-DBT therapist attempts to adopt a dialectical stance that communicates to the patient that weight gain and changes in maladaptive ED behaviors are expected, and yet not sufficient for gaining a life worth living.

#### Skills training

Similar to standard DBT, the function of enhancing capabilities in RO-DBT is translated into the mode of skills training classes (see Table 1 for overview of skills training modules). On an inpatient program, RO-DBT skills training classes are ideally integrated into the daily/weekly program, e.g., daily mindfulness practices (see Table 2 for overview of Haldon unit integrated program). RO-DBT skills are designed to help OC patients learn essential skills to re-join community, including how to engage in novel behavior, express emotions more freely, develop compassion and forgiveness, let go of envy/bitterness, be more playful and spontaneous, activate one’s social-safety system, learn from corrective feedback, and how to form more intimate relationships. Detailed instructor notes, patient handouts, and worksheets are provided in the treatment manual [32].

**Table 1:** RO-DBT and standard DBT skills training modules and targets for Overcontrol and AN-R

**Skills module**	**Behavioral targets**
*Core mindfulness	Rigidity and rule governance
	Imperative of correctness
	Compulsiveness
**Interpersonal effectiveness**	Aloofness and social withdrawal
	Fear of appearing vulnerable
*Emotion regulation	Masking inner feelings
	High social comparison/envy/bitterness
**Distress tolerance**	Self-care neglect
	Rigid needs for structure and order
**Radical openness	Low openness
	Avoiding risk and novelty
	Disregarding feedback
	High distrust and suspicion
	Low empathy/validation of others
	Deficient in forgiveness and compassion

Table Legend: * Indicates that new RO-DBT skills for OC have been incorporated within the standard DBT module. ** Indicates new RO-DBT module for OC that is not part of standard DBT.

**Table 2:** The Haldon unit RO-DBT treatment program

**RO-DBT Functions & Modes**	**Motivating Commitment** (‘structured yet flexible’): via weekly individual therapy & daily monitoring of target behaviors with diary cards
	**Enhancing Capabilities** (‘learning to re-join the tribe’): via skills training classes; 3 RO-DBT skills training classes per week.
	**Skills Generalization** (‘going opposite to stoicism’): via telephone coaching calls as needed when patient off-unit.
	**Support for Therapists** (‘practicing radical openness ourselves’): via weekly RO-DBT staff consultation team meetings.
	**Enhancing Environmental Support**: via RO-DBT informed family therapy sessions emphasizing radical openness and other DBT skills.
**Ancillary groups informed by RO-DBT principles**	**Daily morning mindfulness** and loving-kindness practices.
	**Weekly Target Setting Group:** helping patients ‘look for roses not just thorns’ and practice the art of flexible-planning.
	**Weekly Meal Management Group:** using RO-DBT skills to manage fears of food, urge surfing disgust/bloating sensations, negotiating meal planning using DBT interpersonal effectiveness skills.
	**Weekly Art Group:** exposure to novelty, practicing tolerance of uncertainty, letting go of perfectionism.
	**Weekly Reflective Space:** practicing radical openness skills designed to activate social-safety system; responding without rehearsal.
	**Weekly Body Image Discussion:** observing rigidity and practicing openness to disconfirming feedback.
	**Themed Skills Application Week:** every 2 months the normal Unit schedule is suspended. Staff and patients join together to practice radical openness skills, share community meals, and practice playful spontaneity (e.g., Taiko Drumming, Film Making, Fancy Dress, Pantomime).

##### Radical openness skills

Radical openness skills training encompasses eight separate lessons, usually delivered over a span of eight weeks with weekly homework assignments and handouts/worksheets. As a concept, *radical openness* entails a willingness to surrender prior preconceptions about how the world should be in order to adapt to an ever-changing environment. The practice of radical openness involves three core transacting components: 1) *acknowledgment* or awareness of environmental stimuli that are disconfirming, unexpected, or incongruous, 2) purposeful *self-enquiry* into habitual or automatic response tendencies and emotion-based action urges secondary to the disconfirming feedback (e.g., defend, capitulate, regulate, avoid, accept), and 3) *flexibly-responding* to the feedback by behaving in manner that is genuinely effective in the moment and accounts for the needs of others. Importantly, *radical openness* differs from *radical acceptance* (a core skill in standard DBT [29]). Whereas radical acceptance involves letting go of fighting reality (see [29]; pg. 102), *radical openness challenges our perceptions of reality.* An overview of the weekly skills and key teaching points taught in Radical Openness can be found in Table 3.

**Table 3:** Overview of radical openness skills training module

**Week**	**Skills**
Week 1	Orientation to Radical Openness – Why be radically open?
Week 2	Emotions Communicate to Others—Changing Physiology
Week 3	Engaging in Novel Behavior—Flexible-Mind VARIEs
Week 4	Learning from Corrective Feedback-- Flexible-Mind ADOPTS
Week 5	The Art of Validation—Flexible-Mind Validates
Week 6	Learning to Trust and enhancing Intimacy –Flexible-Mind ALLOWs
Week 7	Developing Compassion and Forgiveness—Flexible-Mind has HEART
Week 8	Increasing Openness & Social Connectedness via Loving-Kindness

Table Legend: Acronyms in the table are used as mnemonic aids. For example, in Week 4, Learning from Corrective Feedback, each letter of the acronym ADOPTS refers to a specific set of skills; A stands for *Acknowledge* that feedback is occurring, D stands for *Describe* and observe emotions, bodily sensations, thoughts, O stands for Open to new information by cheerleading and by fully listening to the feedback, P stands for *Pinpoint* what new behavior is being recommended by the feedback and assess utility, T stands for *Try-out* the new behavior, and S stands for *Self-soothe* and reward yourself for being open to feedback and using skills. Patients are encouraged to memorize acronyms and use them to facilitate memory of skills. Space limitations prevented more descriptive narratives regarding each set of skills and readers are referred to the treatment manual [32].

##### Mindfulness skills

In standard DBT for BPD, mindfulness skills target problems associated with *identity confusion* and *emptiness*[29], whereas OC mindfulness practices target problems associated with *rigid adherence to rules, extreme needs for structure,* and *excessive desires to avoid making mistakes.* Mindfulness practices focus on nonjudgmental acknowledgement of desires for compliance and rule adherence, while cultivating a compassionate, nonjudgmental stance valuing both an *appreciation for rules* and *spontaneity*. Strong OC personal needs for structure are targeted via practices emphasizing nonjudgmental awareness of compulsive urges to fix, organize, correct, or control things whenever a situation is perceived as chaotic, disorganized, uncertain, and/or lacking clarity. Compulsive desires for control are encouraged to be dispassionately observed as inner experiences with predictable action tendencies (action tendencies or urges that are transitory in nature). Participants are encouraged to practice mindful “urge surfing” by gently observing urges to “control, fix, or correct” without getting caught up in the thoughts associated with the urge or mindlessly giving into the action tendencies associated with the urge, i.e., ruminating about a solution, or directing attention toward the problem. Instead, participants are taught to consider the urge like a wave; it crests and then passes [46]. RO-DBT for AN-R teaches ‘urge surfing’ of food-aversive response tendencies, such as sensations of bloating, nausea, urges to vomit, and/or catastrophizing thoughts. Patients are encouraged to dispassionately observe food-aversive response tendencies and are reminded that the practice is similar to techniques used by sailors to overcome seasickness or jet-pilots to overcome severe nausea. Thus, the goal of these practices is not to mindfully enjoy the taste of food; on the contrary, the focus is on noticing aversive sensations/emotions/thoughts associated with food ingestion without responding to them as a crisis^a^. On the Haldon Unit, urge-surfing food-aversive response tendencies represent the only formal mindfulness practice that specifically focuses on food related stimuli. These skills are taught, as needed, in individual therapy and occasionally during group skills classes. Overall, urge surfing is taught and practiced as a general principle for managing aversive sensations/emotions/thoughts that can be used in a wide range of both food or non-food related contexts.

In addition, RO-DBT has new mindfulness “states-of-mind” that represent common OC states that are associated with maladaptive and optimal coping. For OC individuals two states-of-mind are most common and these occur secondary to disconfirming feedback and/or when confronted with novelty. Indeed, when challenged or uncertain, the most common OC response is usually to search for a way to minimize, dismiss, or disconfirm feedback in order to maintain a sense of control and order. This style of behaving in RO-DBT is referred to as *Fixed-Mind.* Fixed-Mind is a problem because it says “change is unnecessary because I already know the answer”. The dialectic opposite of Fixed-Mind is *Fatalistic-Mind*. Whereas Fixed-Mind involves rigid resistance and energetic opposition to change, Fatalistic-Mind involves giving-up overt attempts at resistance. Fatalistic-Mind can be expressed by drawn out silences, bitterness, refusals to participate, and/or sudden acquiescence or a literal suspension of goal-directed behavior and shut-down. Fatalistic-Mind is a problem because it removes personal responsibility by implicating that “change is unnecessary because there is no answer”*.* Mindful awareness of these ‘states’ serve as important skill practice reminders. *Flexible-Mind* forms the synthesis between fixed and fatalistic mind states: it involves being radically open to the possibility of change in order to learn without rejecting one’s past or falling apart. Importantly, although wise-mind in standard DBT [29] and flexible-mind in RO-DBT share some similar functions, there are also important differences. For example, whereas wise-mind celebrates the importance of inner knowing and intuitive knowledge (see [29]; pg. 66), flexible-mind celebrates self-enquiry and encourages compassionate challenges of our perceptions of reality.

##### Emotion regulation skills

In general, emotion regulation skills with OC individuals follow standard DBT protocols (see [29]; pgs. 135–164). Yet, there are some important differences worth noting. First, OC individuals are less likely to exhibit extreme and/or public displays of emotionally dysregulated or impulsive behaviors. Thus, RO-DBT emotion regulation skills target OC tendencies to *mask inner feelings* and emphasize the advantages of experiencing emotions and expressing them when doing so would be effective. Secondly, the primary difference between standard DBT and RO-DBT pertains to new skills targeting *envy, resentment, revenge, and bitterness* stemming from frequent and over-learned tendencies for *social comparison*. High achievement/performance goals, common among OC, necessitate comparison to others in order to determine whether one’s performance (e.g., school grades, body shape) is adequate. Unfortunately, social comparison often results in perceptions of being inadequate or unfairly disadvantaged; experiences that are precursors for *envy and bitterness*. RO-DBT considers unhelpful envy to involve a painful blend of two emotions, shame and anger, with action urges for *secret-revenge.* Opposite emotion action skills for envy focus on going opposite to urges to hide shameful-envious feelings by labeling/revealing them and going opposite to desires for revenge by blocking hyper-vigilance for negative attributes or moral failings of the envied person, blocking pleasurable fantasies of the envied person failing or suffering, and blocking harsh gossip about the envied person. Bitterness is characterized by pessimism, cynicism, and a fatalistic outlook on life; it is a mood state resulting from frequent failures in achieving important goals and/or perceptions that personal success was wrongfully obtained by others. RO-DBT teaches patients to go opposite to bitterness by increasing pro-social behavior, such as practicing giving help/praise and receiving help/praise from others, celebrating successes, resting after completing a difficult task, practicing random acts of kindness and thankfulness for what one has.

##### Distress tolerance skills

Since OC patients are less likely to exhibit impulsive or crisis-oriented behaviors there is less need for crisis survival skills. As a result, only one lesson (one week) is dedicated to teaching distress tolerance skills in RO-DBT skill training classes as opposed to the 6–8 weeks that is typical in standard DBT. Two skills, posited to be particularly helpful for OC patients, are taught during this lesson—self-soothing and radical acceptance skills (see [29]; pg. 167 & pgs. 170–176).

##### Interpersonal effectiveness skills

Most of the interpersonal skills taught in standard DBT [29] are applicable to OC patients. A few modifications in how the skills are taught to OC patients are worth noting. For one, when it comes to interpersonal skill role-plays instructors should be alert to block attempts by some OC patients to “prove they are the best” or to carry out a suggested skills practice that may not be useful simply because the manual suggests it. Secondly, instructors should encourage OC patients to augment interpersonal effectiveness skills practices with Radical Openness skills that are designed to help them enter their neurobiologically based social-safety system (see above).

#### Skills generalization

DBT places a strong emphasis on generalization of treatment gains to *all* physical and emotional contexts. On an inpatient unit this translates into telephone coaching calls with patients away from the unit and/or mini-skills coaching interactions on the unit with staff who are not the primary individual therapist. Although OC patients do experience painful and distressing emotions, they are less likely to express them publicly or engage in dramatic crisis generating displays than UC patients. As a result crisis/coaching calls or requests may be relatively rare among OC patients unless they are encouraged to represent therapeutic progress because they demonstrate willingness to ask for help, lean in for support, and/or display emotional vulnerability—all essential new skills needed by most OC patients.

#### RO-DBT consultation team

As in standard DBT [28], a weekly team consultation meeting is part RO-DBT. Consultation team meetings serve several important functions, including reducing therapist burnout, providing support for therapists, improving phenomenological empathy for patients, and providing treatment planning guidance. This can maximize adherence to the treatment manual. A major assumption in RO-DBT is that that in order to help patients learn to be more open, flexible, and socially connected, therapists must practice the same skills in order to model them to their patients. Thus, the consultation team in RO-DBT is considered an important means by which therapists can practice what they preach.

### Present Study

In the present study we were interested in evaluating an RO-DBT informed inpatient ED service that specialized in the treatment of AN-R. In this ED service, psychological treatment is introduced only after medically unstable individuals have received re-feeding and are medical stable^b^. Our research questions were: (1) What proportion of patients with AN-R completed the treatment (i.e., reaching their agreed weight and therapy goals)? (2) What proportion of patients met criteria for full remission from AN-R, and what proportion met criteria for partial remission at end-of-treatment? We defined “normal” eating disordered behavior as eating behavior that fell within 1 standard deviation of community norms on eating disorder-related pathology. We hypothesized that there would be an increase in the proportion of individuals who met criteria for full and partial remission (3) What were the pre-post treatment changes in BMI and eating disorder psychopathology, psychological functioning and quality of life? We hypothesized that there would be an increase in BMI, psychological functioning and quality of life, and a reduction in psychopathology.

## Methods

### Participants and procedures

All procedures were part of an ongoing service evaluation project approved by the Devon Research and Development Trust. Data was gathered between 1^st^ Jan 2010 and 31^st^ December 2012 as part of routine clinical practice from patients of the Haldon Unit, an inpatient eating disorders Unit within the Devon Partnership Trust in the Southwest of England. The service accepts individuals if they are within the National Health Services (NHS) remit for secondary care, have a mental health condition that is complex enough to warrant a care-coordinator across services and have an eating disorder requiring intensive care within an inpatient medical setting.

#### Inclusion criteria

Individuals were included in the current evaluation if they met ICD-10 diagnostic criteria for Anorexia Nervosa and were primarily restricting-type, as assessed through a clinical interview with the unit psychiatrist at admission. For this first evaluation of RO-DBT it was decided to focus on AN-R because these individuals were posited to most closely resemble the genotypic and phenotypic characteristics the treatment had originally been designed to target. Only the latest admission was entered for patients with multiple admissions to the Haldon Unit between 2010–2012 (23.4% [11/47] of the sample had multiple admissions within this period, max readmissions = 4).

#### Unit intake procedures

**Figure 2:**
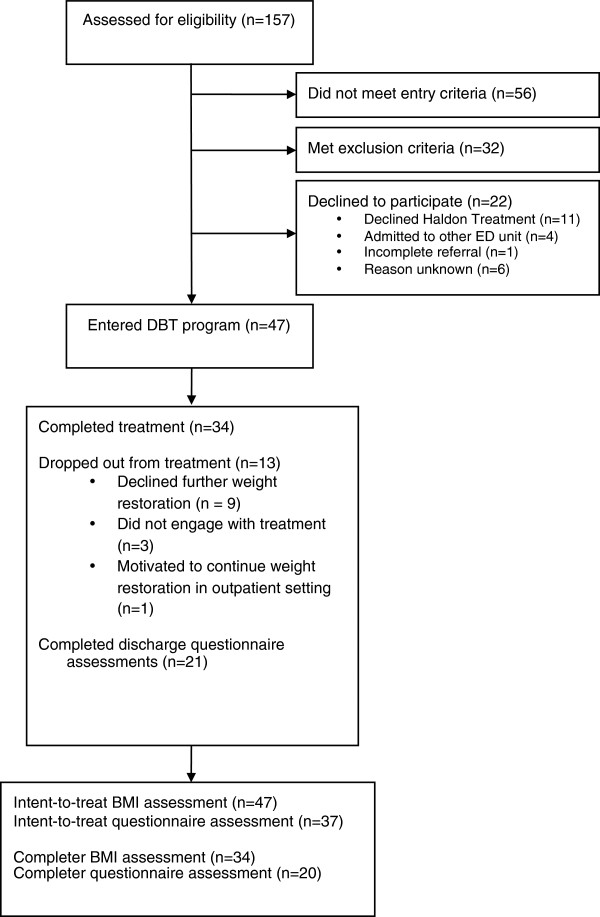
CONSORT flow diagram.

Upon admission to the unit, all patients were given the opportunity to participate in a 2-week Engagement Program designed to familiarize the patient to the RO-DBT program and overall unit structure. During this period, BMI data were obtained and patients were asked to complete a battery of questionnaires, which included the measures utilized in this study. Individuals for whom urgent medical care was a priority first underwent a period of medical stabilization combined with refeeding. Those who met criteria for AN-R were then invited to participate in the RO-DBT program (see Figure 2; Consort Flow Diagram). If patients declined participation in RO-DBT, they were offered to continue the Engagement Program which included a range of psycho-educational groups, specialist support from the multi-disciplinary team, family therapy and mealtime support.

### Treatment setting

Treatment was provided on the Haldon Unit inpatient eating disorder unit in the Southwest of England that is part of the Devon Partnership Trust. Consistent with inpatient eating disorders units across England, the Haldon Unit follows a therapeutic multi-component approach to treatment, which includes psychotherapy, nurse-led care planning, occupational and family therapies, psychiatric consultation, and specialist dietetic counseling. However, the Haldon Unit is unique in that the overall treatment approach on the unit is informed by DBT and RO-DBT principles [28,29] as well as a trans-diagnostic treatment philosophy that accounts for individual differences in self-control tendencies [23]. Individuals with problems of emotional undercontrol (e.g., BN) are treated with standard DBT [28], those with overcontrol (e.g., AN-R) are treated with RO-DBT [32]. The principles of DBT and RO-DBT [28,32] are utilized by staff across each of the treatment modalities, and consultation team meetings are attended by all core staff.

### Radically open-DBT (RO-DBT)

The current study focuses on applying RO-DBT to AN-R. RO-DBT individual therapists were required to be sanctioned by their profession-specific training, licensing, or certification entity to be capable in providing the services associated with individual therapy. For the current evaluation this included psychiatric nurses (n = 11), psychiatrists (n = 3), psychologists (n = 2), dieticians (n = 2), occupational therapists (n = 1), and family therapists (n = 1). All individual therapists were intensively trained by the first author (TL) for 10 days in RO-DBT; support staff who provided skills coaching or assisted with skills training classes received a two-day workshop covering basic principles and an overview of RO-DBT skills (conducted by TL). Clinical supervision of individual therapy was provided during team consultation meetings (weekly by senior staff and monthly by TL). The skills taught during each 8-week cycle (Table 3) were taught continuously and patients could start attending the skills classes at any time during this cycle.

### Measurements

#### Demographic variables

At treatment admission, participants filled in a demographic form, which included information on their gender, age, ethnicity, and number of admissions to date.

#### Body mass index (BMI)

BMI was calculated by the unit’s dietician after measuring the weight and height of each patient.

#### Eating disorder examination-questionnaire (EDE-Q)

The EDE-Q [47] is a 41-item questionnaire adapted from the Eating Disorder Examination [48]; it measures self-reported eating disorder psychopathology. The EDE-Q yields four subscales: Restraint (attempts to restrict food intake), Eating Concern (feelings of guilt and concern about eating), Weight Concern (dissatisfaction with and overvaluation of weight), and Shape Concern (dissatisfaction with and overvaluation of shape). The subscales have good internal consistency (Cronbach’s alphas = .78-.93; [49]) and convergent validity [47]. The community norm for the global EDE-Q in the UK plus 1*SD* is 2.77 [50]. The EDE-Q had good internal consistency in the present study (alpha = .90).

#### Eating disorders quality of life (EDQoL)

The EDQoL [51] is a 25-item questionnaire that measures eating disorder-related quality of life; it consists of the following subscales: Psychological (negative feelings about self), Physical/Cognitive (physical symptoms, including feeling the cold and inability to concentrate), Financial (difficulties with paying bills), and Work/School (needing to take leave/poor performance). The EDQoL has very good internal consistency (alpha = .94), good test-retest reliability (*r* = .93), and good convergent and discriminant validity [51]. The EDQoL had good internal consistency in the present study (alpha = .86).

#### Clinical outcome in routine evaluation (CORE)

The CORE [52] is a 34-item questionnaire measuring the level of psychological global distress a patient has experienced in the last week. It consists of 4 subscales, including Subjective Well-Being (how optimistic vs. overwhelmed a person is feeling), Problems/Symptoms (including anxiety, depression, physical symptoms and trauma), Life Functioning (feelings of loneliness, general coping, and social problems) and Risk/Harm (risk or harm to self or others). It has good internal consistency (.75-.95), test-retest reliability (.87-.91), and good convergent validity [52]. The CORE had good internal consistency in the present study (alpha = .90).

### Study design

Assessments were obtained at treatment admission and end-of-treatment. Questionnaire packs were given to patients by a Clinical Studies Officer or by a nurse.

### Sample size

Previous research on inpatient treatment programs (which focus on weight gain) have reported large effects on BMI from admission to end-of-treatment (i.e. Cohen’s *d* = 1.2 [8]). Thus, with the probability of incorrectly rejecting the null hypothesis set at 0.8, and alpha set at 0.05, a sample size of 26 was considered to be sufficient [53].

### Statistical analysis

Missing admission item scores (<1.5% of the admission item data) were substituted with mean item scores if at least 80% of the questionnaire had been completed. For intent-to-treat analyses we used last observation carried forward (LOCF) because this method has precedent in recent eating disorders research [15] and does not rely on Missing at Random assumptions that become tenuous when data attrition is high. For missing end-of-treatment items (where at least 80% of the end-of-treatment questionnaires had been completed), admission items were carried forward. This was considered to be conservative, as scores on all measures tended to drop over time. “Full remission” was defined as: cessation of severe dietary restrictions^c^, and BMI > 18.5. Similar definitions have been used previously [54]. “Partial remission” was defined as meeting either of these two criteria. Additionally, for ease of comparison with other treatments, we calculated the number of individuals who, on end-of-treatment, had a score on the EDE-Q global subscale that was less than 1*SD* above UK community norms (i.e. <2.77; [50]); we also categorized those who met this criterion in addition to having a BMI > 18.5. Changes in BMI and psychological variables from admission to end-of-treatment were assessed using paired two-tailed *t*-tests, with the alpha-level set at 0.05. Effect sizes were calculated using Cohen’s *d*[55] with 95% confidence intervals.

## Results

### Participant characteristics

Between January 2010 and December 2012, 47 individuals (45 female; mean age = 27.21, *SD* = 10.0) who entered the Unit met eligibility criteria and agreed to participate in the RO-DBT program (see Table 4 for participant characteristics). On admission to the unit they had a mean BMI of 14.22 (*SD* = 1.38). A large proportion of the sample was White British (93.6%), and 39% had been previously admitted to an inpatient unit (of those, 11.2% had been admitted to an inpatient unit 4 or more times previously).

**Table 4:** Participant characteristics

	**Treatment completers**		**Treatment non-completers**		**Comparison between completers and non-completers**
	**(n=34)**		**(n=13)**		
	n	%	n	%	*X*^2^ for frequency variables, independent *t*-tests for continuous variables
Female	32	94.1	13	100	*p*=.37
Ethnicity					
White British	32	94.1	12	92.3	*p*=.25
No answer	2	5.9	1	7.7	
Number of Admissions					
1	18	52.9	9	69.2	*p*=.86
2	8	23.5	2	15.4	
3	3	8.8	1	7.7	
4	2	5.9	1	7.7	
5	2	5.9			
6					
7	1	2.9			
	Mean	*SD*/range	Mean	*SD*/range	
Age (years)	29.65	12.33	24.77	7.66	*p*=.11
		(17-64)		(17-43)	
Admission BMI	14.69	1.49	13.75	1.26	*t*(45)=2.02, *p*=.049
		(12-17.8)		(11.5- 15.8)	
Discharge BMI	18.26	2.18	16.01	1.27	*t*(45)=4.39, *p*<.001
		(15-23)		(15- 18)	

Table Legend: BMI = Body Mass Index.

### What proportion of patients with AN-R completed the treatment?

Of the individuals that met eligibility criteria, 27.7% (13/47) dropped-out of RO-DBT. Drop-out was defined as any instance where an individual discharged themselves from the Unit without agreement from the treatment team that they were ready to leave. The mean number of weeks of treatment was 21.7 for treatment completers (n = 34; range = 3–53) and 13.69 for non-completers (n = 13; range = 4–25). The only pre-treatment difference between the completer and non-completer groups was admission BMI, with those in the completer group having a significantly higher BMI than those in the non-completer group. Treatment non-completers also showed significantly less improvement in mean BMI compared to completers at discharge (see Table 4).

### What proportion of patients with AN-R met criteria for remission?

There were 34 individuals who completed the adapted DBT treatment. All of these individuals provided admission and discharge BMI data, whereas 20 individuals provided data for the psychological variables (via questionnaire packs) at both admission and discharge. For those who completed the RO-DBT treatment and provided both admission and discharge questionnaire data, 35% (n = 7/20) were in full remission, and an additional 55% (n = 11/20) were in partial remission, while only two individuals had a score of >3 on the Restraint subscale of the EDE-Q in addition to a BMI of <18.5. Regarding psychological outcomes, 55% (n = 11/20) had a post-treatment score on the global subscale of the EDE-Q within 1*SD* of community norms (i.e. <2.77). In addition, 30% of patients (6/20) had a score on the global subscale of the EDE-Q that was within 1*SD* of community norms (i.e. < 2.77) in addition to a BMI ≥18.5 (see Table 5 for means and *SD* of all scales).

**Table 5:** RO-DBT completer analyses

		**Admission**			**Discharge**			**Change score**	***p*-value**	**Effect size**
		***M***	***SD***	***n***	***M***	***SD***	***n***			**Cohen’s *d *** **(95% CI)**
BMI		14.69	1.49	34	18.26	2.18	34	3.57	<.001	1.91 (1.32-2.49)
EDE-Q	Global	4.34	1.16	20	2.81	1.44	20	1.53	<.001	1.17 (0.57-1.75)
	Restraint	3.97	1.66	20	1.66	1.75	20	2.31	<.001	1.35 (0.69-1.99)
	Eating Concerns	3.95	1.07	20	2.37	1.37	20	1.58	<.001	1.29 (0.64-1.91)
	Shape Concerns	4.85	1.35	20	4.15	1.44	20	0.70	=.028	0.50 (0.05-0.94)
	Weight Concerns	4.59	1.54	20	3.07	1.70	20	1.52	<.001	0.94 (0.43-1.42)
EDQoL	Global	2.11	0.68	14	1.44	0.62	14	0.67	=.003	1.03 (0.35-1.68)
	Psychological	3.15	0.54	20	2.12	0.76	20	1.03	<.001	1.56 (0.92-2.19)
	Physical/Cognitive	2.60	0.67	20	1.13	0.85	20	1.47	<.001	1.92 (0.12-2.70)
	Finance	0.78	0.94	18	0.59	1.05	18	0.19	=.351	0.19 (−0.21-0.58)
	Work	1.65	1.13	14	1.38	0.97	14	0.27	=.579	0.26 (−0.63-1.14)
CORE	Global	2.24	0.53	20	1.46	0.63	20	0.78	<.001	1.34 (0.68-1.98)
	Well-being	3.16	0.60	20	2.20	0.86	20	0.96	=.001	1.29 (0.57-2.00)
	Problems	2.82	0.60	20	1.83	0.77	20	0.99	<.001	1.43 (0.70-2.15)
	Functioning	2.30	0.59	20	1.41	0.58	20	0.89	<.001	1.52 (0.86-2.16)
	Risk	0.73	0.78	20	0.43	0.61	20	0.30	=.035	0.43 (0.03-0.82)

Table Legend: BMI = Body Mass Index; EDE-Q = Eating Disorder Examination Questionnaire;

EDQoL = Eating Disorder Quality of Life; CORE = Clinical Outcome in Routine Evaluation.

For the intent-to-treat sample, 20.5% (n = 8/39) were in full remission, and an additional 41.0% (n = 16/39) were in partial remission, while the remaining 15 individuals had a score of >3 on the Restraint subscale of the EDE-Q in addition to a BMI of <18.5. Regarding psychological outcomes, 35.9% (n = 14/39) had a post-treatment score on the global subscale of the EDE-Q within 1*SD* of community norms (i.e. <2.77). Lastly, 20.5% of patients (8/39) had a score on the global subscale of the EDE-Q that was within 1*SD* of community norms (i.e. < 2.77) in addition to a BMI ≥18.5 (see Table 6 for means and *SD* of all scales).

**Table 6:** Intent-to-treat analyses

		**Admission**			**Discharge**			**Change score**	***p*-value**	**Effect size**
		***M***	***SD***	***n***	***M***	***SD***	***n***			**Cohen’s *d *** **(95% CI)**
BMI		14.43	1.48	47	17.64	2.21	47	3.21	<.001	1.71 (1.25-2.16)
EDE-Q	Global	4.44	1.09	37	3.53	1.49	37	0.91	<.001	0.70 (0.35-1.04)
	Restraint	4.12	1.65	37	2.64	2.10	37	1.48	<.001	0.78 (0.41-1.15)
	Eating Concerns	4.03	1.06	37	3.11	1.53	37	0.92	<.001	0.70 (0.34-1.05)
	Shape Concerns	4.98	1.21	37	4.60	1.33	37	0.38	=.026	0.30 (0.03-0.56)
	Weight Concerns	4.62	1.35	37	3.77	1.63	37	0.85	<.001	0.57 (0.25-0.87
EDQoL	Global	2.06	0.55	26	1.69	0.64	26	0.37	=.004	0.62 (0.19-1.04)
	Psychological	3.17	0.48	37	2.54	0.86	37	0.63	<.001	0.90 (0.50-1.30)
	Physical/Cognitive	2.61	0.70	37	1.86	1.16	37	0.75	<.001	0.78 (0.36-1.19)
	Finance	0.73	0.85	33	0.59	0.92	33	0.14	=.205	0.16 (−0.09-0.40)
	Work	1.67	0.97	27	1.62	0.83	27	0.05	=.815	0.06 (−0.40-0.51)
CORE	Global	2.21	0.53	36	1.75	0.74	36	0.46	<.001	0.71 (0.31-1.11)
	Well-being	3.06	0.65	36	2.51	0.87	36	0.55	=.001	0.72 (0.29-1.13)
	Problems	2.80	0.57	36	2.21	0.85	36	0.59	=.001	0.82 (0.35-1.27)
	Functioning	2.27	0.60	36	1.75	0.78	36	0.52	<.001	0.75 (0.34-1.14)
	Risk	0.72	0.74	36	0.55	0.77	36	0.17	=.063	0.23 (−0.01-0.46)

Table Legend: BMI = Body Mass Index; EDE-Q = Eating Disorder Examination Questionnaire;

EDQoL = Eating Disorder Quality of Life; CORE = Clinical Outcome in Routine Evaluation.

### Change in weight

#### BMI

For the treatment completers, there was a large and significant difference from admission to discharge corresponding to a large effect size on patient BMI (*d =* 1.91, see Table 5). In the intent-to-treat analyses, there was also a significant increase in mean BMI from admission to discharge corresponding to a large effect size (*d =* 1.71, see Table 6).

### Eating disordered psychological functioning

As can be seen in Table 5, there was a large overall reduction on global EDE-Q scores between admission and discharge in those who completed the questionnaires at admission and end-of-treatment. There was also a significant reduction from admission to discharge on the Restraint subscale, the Eating Concerns subscale, the Shape Concerns subscale and the Weight Concerns subscale. The intent-to-treat sensitivity analyses also demonstrated a large and significant improvement in Restraint, and Eating Concern EDE-Q scores from admission to end-of-treatment, and a medium and significant improvement in global EDE-Q and Weight Concerns. In the intent-to-treat analyses, Shape Concerns improved significantly, but the corresponding effect size was small (see Table 6).

### Eating disorder quality of life

Global EDQoL scores were computed for those who answered all subscales. A number of patients did not answer the Work and Finance subscales of the EDQoL, as many individuals were not employed (see Table 5). In questionnaire completers, scores on the EDQoL improved from admission to discharge (note, a higher score on the EDQoL is representative of a *lower* QoL). There was a large overall reduction on the global EDQoL scores between admission and discharge, indicating increased QoL. There was a significant decrease in EDQoL scores from admission to discharge on the Psychological subscale, the Physical/Cognitive subscale, but not on the Finance or Work subscales (see Table 5). For the intent-to-treat analyses, there were significant improvements in global EDQoL, and the Psychological and Physical/Cognitive subscales, all corresponding to large effect sizes. There were no significant improvements in the Finance or Work subscales (see Table 6).

### Distress

Global CORE scores in questionnaire completers significantly reduced from admission to discharge (high scores = greater dysfunction). There were significant decreases in CORE scores for the Subjective Well-Being, Problems/Symptoms, Life Functioning and Risk/Harm subscales (see Table 5). In the intent-to-treat analyses, there were likewise significant and large improvements in global CORE scores, and the Well-Being, Problems/Symptoms and Life Functioning subscales. There were no significant improvements in the Risk subscale (see Table 6).

## Discussion

The primary aim of this study was to obtain preliminary data on the feasibility and outcomes of a novel adaptation of DBT (RO-DBT) targeting overcontrol (OC) that was integrated into a comprehensive inpatient treatment program for adult AN-R. Although the efficacy of RO-DBT has been established for refractory depression and comorbid OC personality disorders [34,35] this is the first systematic evaluation of the new treatment with AN-R. Importantly, RO-DBT provides a unique perspective on the etiology underlying AN (specifically the restrictive subtype) by conceptualizing restrictive eating as a form of maladaptive inhibitory control that is part of an overcontrolled style of coping [18,32].

There were three main findings from this study. The first is that RO-DBT is a feasible treatment for individuals suffering from AN-R delivered in inpatient settings. Compared with drop-out rates of 13-66% in adults with chronic AN [56], only 27.7% of individuals in the current study dropped-out of treatment. This is notable, given the severity of the patients in the current study (e.g., mean admission BMI = 14.43, *SD* = 1.48).

Second, the results of the analyses of weight gain suggest that RO-DBT is a promising treatment for AN-R. Intent-to-treat (ITT) analyses demonstrated significant improvements in weight; despite the fact that RO-DBT does not emphasize the importance of targeting ED behaviors or weight gain and instead focuses on obtaining a life worth living. This contrasts sharply with other ED treatments, including standard DBT for undercontrolled binge-purge problems [30,31] that consider eating disordered pathology to take priority over other quality-of-life targets.

The increase in BMI in the ITT analyses was equivalent to a large effect size of *d* = 1.71, which contrasts with an effect size of *d* = 1.2 reported for other inpatient programs [8]. ITT analyses also revealed 20.5% of the sample to be in full remission and 41.0% in partial remission, with higher rates among those completing treatment (35% in full remission and 55% in partial remission). These rates of remission are encouraging, as literature on AN recovery has demonstrated that higher BMI attainment in treatment predicts better relapse prevention [57,58]. Furthermore, these remission rates are comparable to those achieved in outpatient settings, and are noteworthy because they were achieved in a more severely underweight and chronic population.

Third, consistent with recommendations that studies of AN should assess changes in quality of life and psychological functioning [56], we found that individuals who responded to the questionnaires demonstrated significant improvements (all large effect sizes) in both general psychological distress and well-being, and in eating disordered quality of life. ITT analyses paralleled these findings showing significant changes in global eating disorder pathology, global quality of life, and global level of distress. However, for both completer and ITT analyses there were no significant changes in work or finance quality of life which may be due to the patient being in the hospital during the assessment. Global improvement in well-being among hospitalized AN patients is important given the high relapse rates common among inpatients which may reflect a general lack of change in psychological functioning [59].

The findings are important because the treatment examined in this study is fundamentally different from most other approaches. For one, most inpatient units undertake a multicomponent, theoretically eclectic approach. This study is one of the first to examine an inpatient unit where a unified treatment philosophy was fully integrated across each treatment modality (e.g., psychological therapy, medical, occupational therapy, massage therapy, dietetics). Second, the treatment approach strongly emphasizes the importance of learning new skills to enhance flexible responding that can be translated across settings (inpatient to outpatient) without requiring support from the same therapist. Indeed, efficacy studies of RO-DBT have been conducted in outpatient settings (for a review, see [36]) suggesting the utility of integrating the treatment into outpatient programs in order to address differing levels of severity. Thirdly, the RO-DBT model is trans-diagnostic in nature; self-control tendencies are hypothesized to exhibit quadratic (inverted-U) relations with psychological well-being with either extreme of overcontrol or undercontrol predicted to be treatment-resistant. This has clear treatment implications. Undercontrolled problems require interventions designed to enhance inhibitory control, whereas overcontrolled problems require interventions designed to relax inhibition and promote flexible responding [36]. Thus, although not an explicit aim of the current study, by incorporating both standard DBT and RO-DBT into the overall treatment package, the Haldon unit provides a template for addressing both undercontrolled and overcontrolled eating disordered problems within one framework.

### Limitations and future directions

This unfunded study focused on gathering preliminary data and given this has three main limitations. First, the study lacked post-treatment follow-up data. Although we have attempted to gather this data, we have been limited by structural barriers in the UK healthcare system that have limited our ability to contact participants. Therefore, it is not possible to ascertain the extent improvements are retained over time. We have, however, used these barriers as an opportunity to modify our follow-up procedures with new patients in the unit. Second, although we were able to gather BMI data for the entire sample, there was less questionnaire data available (79% of the intent-to-treat sample completed their questionnaires). Both of these issues reflect the lack of external grant support for the current evaluation suggesting the importance of future studies to develop methods for obtaining these data.

Third, as might be expected, amount of weight gain achieved differed between treatment completers and non-completers. In the overall sample, average BMI at discharge was 17.64 while in treatment completers average BMI at discharge was 18.26. This suggests that individuals who completed treatment were more likely to no longer meet the weight criteria for AN. These outcomes were also reflected in rates of remission, albeit those dropping-out of treatment also had lower admission BMIs. This may reflect the more severe nature of their AN, although it may also point to the need to encourage inpatient admission at an earlier point in outpatient treatment.

Future research should examine RO-DBT with both AN restricting and AN binge-purge types, particularly since DBT has already shown efficacy in the treatment of bulimia and binge-purge problems (e.g., see [60] for a review). In addition, the skills training focus that is inherent in DBT provides a unique means for generalizing treatment gains from inpatient to outpatient settings, without depending solely on the establishment of a strong therapeutic relationship as a basis for change. Future studies should also examine stepped-care approaches, particularly given the ego-dystonic and medically risky nature of AN which may necessitate approaches that account for both motivational problems, as well as the possibility of hospitalization. Importantly, the Haldon unit represents a unique type of therapeutic community which discourages arbitrary boundaries between staff and patients. Therapists are encouraged to *practice what they preach*, thereby creating an ethos that values skills usage and self-enquiry whilst signaling to the hyper-perfectionistic AN patient a message that all humans share a common bond of fallibility. Interestingly, since implementing this approach, anecdotal reports from management have noted a significant reduction in staff sick-leave and increases in work satisfaction. This suggests the importance of examining the cost and health benefits associated with differing treatment philosophies on staff retention, burn-out, and sick leave, factors that may be important moderators of treatment outcome.

## Conclusion

The findings from this preliminary evaluation of a novel adaption of DBT applied to AN-R are promising. RO-DBT provides an original perspective regarding the etiology and treatment of AN via a biosocial model that accounts for temperamental, family/environmental, perceptual, and self-control tendencies. Restrictive and ritualized eating is conceptualized as a type of maladaptive self-control that has been intermittently reinforced. Moreover, treatment strategies focus less on food related issues and more on principles posited important for emotional well-being, including openness to new or disconfirming feedback, flexible-responding to changing environmental demands, and recognition that emotions evolved to communicate [61] thereby highlighting the importance of *social signaling* in forming close interpersonal bonds. Finally, the study design follows recommendations that new treatments for AN undergo preliminary testing prior to the conduct of a randomized trial [22,56] and the strength of the results support the utility of further testing via randomized controlled trials.

## Endnotes

^a^RO-DBT for AN-R does not require the development of an exposure hierarchy of forbidden foods when teaching urge-surfing. Instead, urge-surfing is taught as a general principle for managing aversive sensations/emotions/thoughts that can be used in a wide range of contexts. That said, individual therapists are encouraged to utilize hierarchical exposure techniques—if collaboratively deemed useful for a particular patient.

^b^We recognize that there is debate about the appropriateness of psychological interventions while the individual is medically unstable and in the process of refeeding. However, we note that the intervention under investigation was implemented only following medical stabilization, as the poor cognitive functioning of a medically unstable individual may not be an appropriate time in which to introduce a psychological intervention.

^c^Operationalized as ≤3 on all of the first 5 items of the EDE-Q (Restraint subscale).

## Abbreviations

DBT: Dialectical behavior therapy; RO-DBT: Radically open-DBT; OC: Overcontrol.

## Competing interests

TRL-receives royalties from Guilford Press from the book written on RO-DBT.

## Authors’ contributions

TRL is the developer of the treatment and underlying theory examined in this study. He initiated the design of the study and provided oversight of data collection. He provided oversight on data analyses and was seminal in taking a lead role in writing the final manuscript. KLHG was responsible for and conducted the primary data analyses for the study, she interfaced with Unit staff to ensure accurate data collection, and she also helped draft the manuscript. RJH was responsible for setting up data collection procedures on the Unit and ensuring accurate data collection; she contributed to the data analyses, and provided critical revisions to improve the intellectual content of the manuscript. MT was responsible for ensuring data collection, provided clinical leadership regarding the new treatment on the Unit, and helped draft the manuscript. EYC contributed to the draft of this manuscript and critically revising it by providing important intellectual content. HAO contributed to the data analyses, provided critical oversight over data collection procedures, and helped draft and critically evaluate the writing of the manuscript. All authors read and approved the final manuscript.

## Pre-publication history

The pre-publication history for this paper can be accessed here:

http://www.biomedcentral.com/1471-244X/13/293/prepub
